# Atypical Presentation of PKDL due to *Leishmania infantum* in an HIV-Infected Patient with Relapsing Visceral Leishmaniasis

**DOI:** 10.1155/2014/370286

**Published:** 2014-08-14

**Authors:** Benedetto Maurizio Celesia, Bruno Cacopardo, Daniela Massimino, Maria Gussio, Salvatore Tosto, Giuseppe Nunnari, Marilia Rita Pinzone

**Affiliations:** ^1^Department of Clinical and Molecular Biomedicine, Division of Infectious Diseases, University of Catania, Via Palermo 636, 95125 Catania, Italy; ^2^Department of Clinical and Molecular Biomedicine, Unit of Dermatology, University of Catania, 95100 Catania, Italy

## Abstract

We describe the case of an Italian patient with HIV infection who developed an atypical rash resembling post-kala-azar dermal leishmaniasis (PKDL) when receiving liposomal Amphotericin B (L-AMB) for secondary prophylaxis of visceral leishmaniasis (VL). At the time of PKDL appearance, the patient was virologically suppressed but had failed to restore an adequate CD4+ T-cell count. Histology of skin lesions revealed the presence of a granulomatous infiltrate, with lymphocytes, plasma cells, and macrophages, most of which contained *Leishmania* amastigotes. Restriction fragment length polymorphism-polymerase chain reaction was positive for *Leishmania infantum*. Paradoxically, cutaneous lesions markedly improved when a new relapse of VL occurred. The patient received meglumine antimoniate, with a rapid clinical response and complete disappearance of cutaneous rash. Unfortunately, the patient had several relapses of VL over the following years, though the interval between them has become wider after restarting maintenance therapy with L-AMB 4 mg/kg/day once a month. Even if rare, PKDL due to *Leishmania infantum* may occur in Western countries and represents a diagnostic and therapeutic challenge for physicians. The therapeutic management of both PKDL and VL in HIV infection is challenging, because relapses are frequent and evidence is often limited to small case series and case reports.

## 1. Introduction

Leishmaniasis is a vector-borne disease caused by protozoans from the genus* Leishmania*, which are typically spread through the bite of infected female phlebotomine sandflies. Leishmaniasis is characterized by a spectrum of clinical manifestations including cutaneous leishmaniasis, mucosal leishmaniasis, and visceral leishmaniasis (VL) [1–4]. Post-kala-azar dermal leishmaniasis (PKDL) is a dermatosis that usually occurs as a complication of VL [5]. It is characterized by a macular, maculopapular, and nodular rash, generally appearing on the face and eventually spreading to the arms and chest. Most cases have been described in the Indian subcontinent and Sudan, where* Leishmania* (L.)* donovani *is the causative agent [5]. However, PKDL has been rarely associated with* L. infantum *[6–10]. Though VL is a common opportunistic disease in HIV-positive patients, only few cases of PKDL in the setting of HIV infection have been reported so far [6–10].

Here we describe the case of a 33-year-old HIV-infected patient who developed an unusual rash resembling PKDL when receiving liposomal Amphotericin B (L-AMB) for secondary prophylaxis of VL.

## 2. Case Report

**Figure 1:**
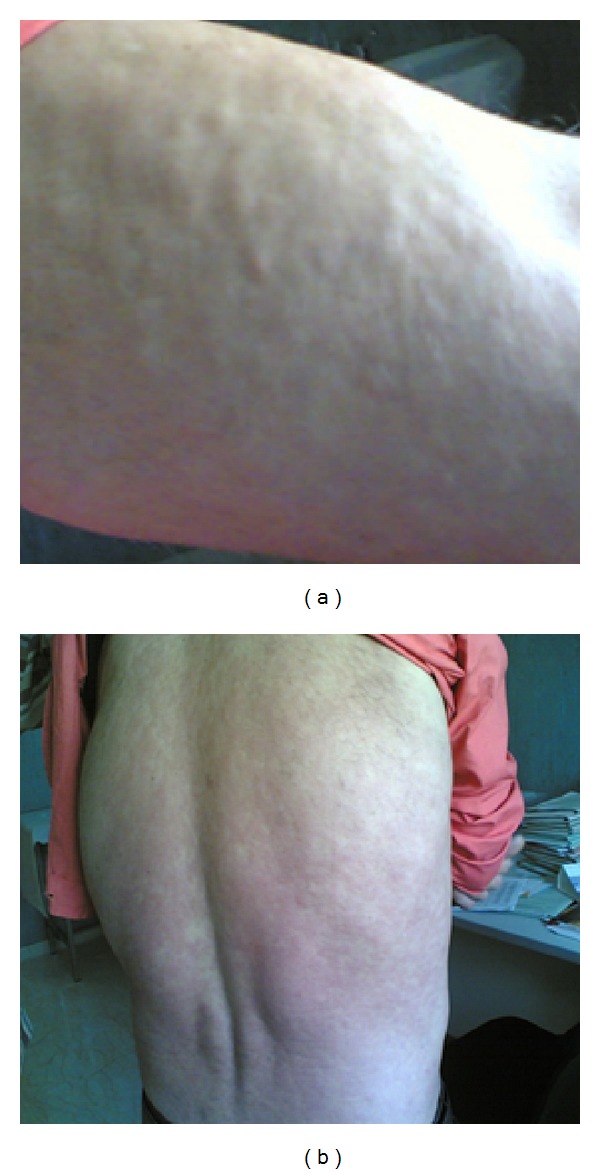
Diffuse maculopapular and nodular rash on the upper limbs (a) and trunk (b).

In May 2002, a 33-year-old Italian man presented to our outpatient clinic complaining of fever, weight loss, and diarrhea. He had mucocutaneous lesions highly suggestive of Kaposi's sarcoma (KS). On physical examination he had hepatosplenomegaly; laboratory tests showed pancytopenia and polyclonal hypergammaglobulinemia. He tested HIV positive. His CD4+ T-cell count was 17 cells/*μ*L and HIV RNA viral load was 225,181 copies/mL. A bone marrow smear showed the presence of* Leishmania* spp. amastigotes. As a consequence, diagnosis of VL was made and treatment with meglumine antimoniate at a dose of 20 mg/kg/day was given for 30 days. Moreover, HAART with Lamivudine-Stavudine-Lopinavir/ritonavir was started. The patient clinically improved, with progressive resolution of KS lesions and good virological response to antiretroviral therapy. Six months later, the patient suffered a clinical relapse of VL and was treated with intravenous liposomal Amphotericin B (L-AMB) at a dose of 4 mg/kg/day for 5 days and then weekly at the same dose for 4 weeks. However, several relapses of VL occurred over the following years. Each relapse was treated with the aforementioned drug schedule. In February 2008, it was decided to start maintenance therapy with L-AMB at a dose of 4 mg/kg/day once a month, in an attempt to prevent further relapses. At this time, his HAART regimen consisted of Abacavir-Lamivudine-Lopinavir/ritonavir; HIV viral load was stably below 50 copies/mL and CD4+ T-cell count was 125 cells/*μ*L. Some weeks after starting secondary prophylaxis with L-AMB, a maculopapular and nodular rash appeared on his trunk and upper limbs (Figure 1). The rash gradually worsened and, four months later, treatment with L-AMB was stopped and a cutaneous biopsy was performed. Histology revealed the presence of a granulomatous infiltrate, with lymphocytes, plasma cells, and macrophages, most of which contained* Leishmania* amastigotes. Restriction fragment length polymorphism-polymerase chain reaction (RFLP-PCR) was positive for* L. infantum*. These findings were consistent with a diagnosis of PKDL. In September 2008, the patient started complaining of fever, diarrhea, and night sweats. Paradoxically, cutaneous lesions markedly improved. On the basis of his symptoms, a relapse of VL was suspected and confirmed by bone marrow aspirate, which showed the presence of* Leishmania* amastigotes. Treatment with meglumine antimoniate was restarted at a dose of 20 mg/kg/day for 30 days. The patient experienced a rapid clinical response and complete disappearance of cutaneous rash. Unfortunately, the patient had several relapses of VL over the following years, though the interval between them has become wider after restarting maintenance therapy with L-AMB 4 mg/kg/day once a month. At the last followup in December 2013, the patient was in good health and was still receiving secondary prophylaxis with L-AMB. He was taking HAART with Abacavir-Lamivudine-Lopinavir/ritonavir-raltegravir with his last CD4+ T-cell count being 98 cells/*μ*L and HIV RNA < 20 copies/mL.

## 3. Discussion

PKDL usually occurs in patients with a previous history of VL living in endemic areas, such as Eastern Africa and the Indian subcontinent, where* L. donovani* is the causative parasite [5]. Only few reports have associated PKDL with* L. infantum*, mostly in HIV-infected subjects [6–11]. PKDL usually manifests as maculopapular or nodular lesions on the face, trunk, and limbs. In our case, the patient developed an atypical rash that spared his face. Of interest, skin lesions occurred when he was receiving secondary prophylaxis with L-AMB and significantly improved when a relapse of VL occurred. It may be hypothesized that antileishmanial treatment could have forced parasites to seek refuge in the dermis, delaying the relapse of VL but not PKDL.

The pathogenesis of PKDL is still partially unclear. PKDL probably represents the attempt of the immune system to clear latent dermal parasites. In HIV-infected subjects, PKDL may represent a clinical manifestation of immune reconstitution inflammatory syndrome (IRIS). In fact, some authors reported PKDL to occur in patients experiencing immune recovery after the introduction of HAART [6–10]. It seems not to be the case in our report, as the patient developed PKDL several years after starting antiretroviral therapy and had a poor CD4+ T-cell recovery, despite the persistently undetectable HIV viral load. Failure to restore adequate CD4+ T-cell responses may explain the inefficiency of antileishmanial treatment and the high number of relapses of VL [1]. The course of VL in HIV infection is associated with a lower treatment response rate and a higher incidence of relapses and mortality than in the general population. In a recent meta-analysis, lack of recovery of CD4+ T cells after VL has been found to predict relapses, along with lack of secondary prophylaxis, previous history of relapses, and a CD4+ T-cell count under 100/*μ*L at the time of primary VL diagnosis [12].

T-helper (Th)1 cytokines, such as Interleukin- (IL-) 12 and Interferon-*γ*, have a crucial role in the promotion of parasite killing [13]. HIV infection favours a Th2 shift, especially in the advanced stages of the disease, which is detrimental to leishmaniasis [14]. In fact, increased production of Th2 cytokines, in particular IL-10, has been associated with higher risk of* Leishmania* visceralization [15]. Unfortunately, we could not evaluate the immunological profile of our patient in order to define the Th1/Th2 balance at the time of PKDL and VL relapses.

The optimal treatment of PKDL in HIV-infected patients is unknown, as most data come from case reports. Pentavalent antimonials [7–10], liposomal amphotericin or other lipid formulations [6–10], pentamidine [6, 7], and miltefosine [6, 8, 10] have been used with mixed results. As for secondary prophylaxis, it seems advisable to use maintenance therapy to prevent further relapses, at least until CD4+ T-cell count falls below 200 cells/*μ*L [1]. Different regimens with several drugs have been used, including pentavalent antimonials (20 mg/kg/day given every 3 to 4 weeks), Amphotericin B (either L-AMB or amphotericin lipid complex) (3 to 5 mg/kg/day given every 3 to 4 weeks), and pentamidine (4 mg/kg/day given every 3 to 4 weeks), but there are no well-established protocols [1]. In a small study, miltefosine was used for secondary prophylaxis of VL in HIV-infected subjects at a dose of 50 mg/day given 3 times a week [16]. However, prospective studies are needed to establish the optimal therapeutic approach to relapsing VL.

In conclusion, our report shows that even if rare PKDL due to L.* infantum* may occur in HIV-positive patients and it should be included in the differential diagnosis of skin lesions, especially if the patient has a history of VL or he is from or has traveled to an endemic area. The therapeutic management of both PKDL and VL in the setting of HIV infection is challenging, as data are limited, with most evidence coming from small case series and case reports. Randomized controlled trials are warranted to establish the most appropriate approach to* Leishmania*/HIV coinfection.
